# Supplementary material for: Curability difference between autochthonous mouse tumors and their transplants in association with immune gene expression
Source: PLoS One. 2026 Apr 24;21(4):e0338289. doi: 10.1371/journal.pone.0338289 (PMC13108875; doi:10.1371/journal.pone.0338289)
Supplement: S1 Fig — (DOCX) [file pone.0338289.s001.docx]

**S1 Fig**

**S1 Fig.** **Curability difference between 3MC-induced autochthonous tumors and their transplants.** Tumors were irradiated with a collimated 6MV X-ray beam when grown to a size of 0.8-1 cm in diameter. Autochthonous tumors regressed once to the non-palpable size after irradiation, as did transplanted tumors; however, most of them reappeared during 120 days post-irradiation. Curability increased in a dose dependent manner for transplanted tumors (■), while most of autochthonous tumors were incurable (◆). The small peak in the lower curve indicates the presence of benign tumors, as the A1 tumor in the present study, among autochthonous tumors. The dose response curves were re-plotted from numerical data in Ref. 4.
