# Supplementary material for: Curability difference between autochthonous mouse tumors and their transplants in association with immune gene expression
Source: PLoS One. 2026 Apr 24;21(4):e0338289. doi: 10.1371/journal.pone.0338289 (PMC13108875; doi:10.1371/journal.pone.0338289)
Supplement: S2 Fig — (DOCX) [file pone.0338289.s002.docx]

**S2 Fig**

**A Liver B Lung**

**S2 Fig. Immune gene expressions in the mouse liver and lung as measured by qPCR.** **A.** Liver of mouse carrying the A1 tumor (A1), livers of mice carrying A2-A12 tumors (A: n = 11), and livers of control female C57BL/6J mice (C: n = 2). Differences are statistically not significant for all (*p* > 0.05). **B.** Lung of mouse carrying the A1 tumor (A1), lungs of mice carrying A2-A12 tumors (A: n = 11), and lungs of control female C57BL/6J mice (C: n = 4). _*_ : statistically significant, *p* < 0.05.
