# Supplementary material for: Curability difference between autochthonous mouse tumors and their transplants in association with immune gene expression
Source: PLoS One. 2026 Apr 24;21(4):e0338289. doi: 10.1371/journal.pone.0338289 (PMC13108875; doi:10.1371/journal.pone.0338289)
Supplement: S1 File — (DOCX) [file pone.0338289.s003.docx]

**Protocols**

Curability Difference between Autochthonous Mouse Tumors and Their Transplants in Association with Immune Gene Expression. H. Tanooka, C. Kudo-Saito, F. Chiwaki, M. Ishiai, K. Tatsumi, H. Sasaki, T. Ochiya

**Mice**

All animal experiments were approved by the National Cancer Center (T21-006) and conducted in accordance with the Guidelines for Animal Experiments in the National Cancer Center and the 3Rs stipulated by the Animal Welfare Management Act.

Specific pathogen free (SPF) female C57BL/6J mice (PPID: IMSR_IAX:000664) at 10 weeks of age were purchased from Charles River Japan and kept in the SPF room with standard animal diet and water *ad libitum*. A maximum of four mice were housed per cage.

**Tumor induction and transplantation**

An aliquot of 0.1 ml of 3-MC (Sigma) dissolved in 5 mg/ml in olive oil (Fujifilm-Wako) was injected into the groin. Tumor formation was monitored by palpation. The first tumor was detected 76 days after MC injection.

When tumors reached 1 cm in diameter, mice were euthanized by cervical dislocation. Tumors were resected and divided into sections for histological examination, transplantation, and gene expression studies.

For transplantation, a tumor fragment was subcutaneously transplanted into the groin of a female C57BL/6J mouse using a 13-gauge transplantation needle (KN391-25, Natsume). Transplantation were performed in duplicate. Transplanted tumors were analyzed as autochthonous tumors.

Liver and lung tissues were collected at the time of tumor resection. All tumors and organs samples were stored at - 80℃ until further analysis.

**Histology**

Tumor specimens were fixed in 10% formalin (Fujifilm-Wako), embedded in paraffin, sliced, stained with hematoxylin and eosin (Sakura Finetek),. Histological evaluation was performed using a microscope (Olympus BX5).

**Immune gene expression measurement with qPCR**

Two sections of each tumor were homogenized in a QIAzol lysis reagent (Qiagen) with a homogenizer (Kinematica). Total RNA was extracted using the miRNeasy kit (Qiagen).

RNA concentration was adjusted to 0.2 μg/μl with nuclease-free water (Qiagen) and 2.5 μl was transcribed in in a 10 μl reaction using the High Capacity cDNA Reverse Transcription kit (Applied Biosystems). The resulting cDNA was diluted 1:10 with nuclease-free water (Qiagen).

Quantitative PCR was performed using TaqMan Fast Advanced Master Mix (Applied Biosystems) and TaqMan Gene Expression Assays for the following targets

*Pd1* (Mm01285676_ml), *Pdl1* (Mm03043248_ml), *Pdl2* (Mm00451734_ml), *Cd3d* (Mm00442746_ml), *Cd8a* (Mm01182107_gl), *Cd8b* (Mm00438116_ml), *ifnγ* (Mm01168134_ml), *Itga2* (Mm00434371_ml), *Gzmb* (Mm00493152_gl), *Foxp3* (Mm0047516_ml), and *β-actin* (Mm00607939_sl) as an internal control.

Each 12.5 μl reaction mixture contained 4 μl diluted cDNA and manufacturer’s recommended concentration of primers and probes. Aliquotes were put on a 96 well PCR plate. Amplification was performed on a CFX90 Real Time PCR System (Bio Rad, SCR 018064) under the following conditions: 95°C for 3 min, followed by 40 cycles of 95°C for 10 s and 55°C for 30 s.

Relative gene expression levels were calculated using the ΔCt method normalized to β-actin. Data processing was performed using Excel (Microsoft; RRID: SCR_017294).

**Immunohistochemical analysis**

Paraffin-embedded tumor sections were stained with the following antibodies: anti-DX5-FITC (BioLegend #108909, RRID:AB_313416), anti-CD8-PE-Cy5 (BD Bioscience #553034, RRID:AB_394572), anti-IFNγ-PE (BD Bioscience #554412, RRID:AB_395376), and the appropriate isotype controls (BD Bioscience).

Three locations per section were observed at 100× magnification using a LSM700 laser scanning confocal microscope (Carl Zeiss, RRID: SCR_020925). The numbers of DX5^+^ NK cells and CD8^+^ T cells per field were manually counted.

The immunofluorescence intensity of IFNγ expression was quantified as pixel counts in three field per section using ZEN 2012 software (RRID:m SCR_013872) installed on the LSM700 system (Carl Zeiss, RRID: SCR_017377).

**Statistics**

Student’s t-test was applied to statistical analysis of measured data, using Excel program (RRID:SCR_017294). A p-value <0.05 was considered statistically significant.
