# Supplementary material for: Curability difference between autochthonous mouse tumors and their transplants in association with immune gene expression
Source: PLoS One. 2026 Apr 24;21(4):e0338289. doi: 10.1371/journal.pone.0338289 (PMC13108875; doi:10.1371/journal.pone.0338289)
Supplement: Attachment — Submitted filename: Response to Journal Requirements.docx [file pone.0338289.s006.docx]

**Response to Journal Requirements #3 sent by Ms. Wagan on March 5, 2026**

1. We appreciate your assistance in removing the unnecessary older documents. We have reviewed the list of documents accordingly.
2. **Figure legends:** The figure legends have been placed at the end of the main text after the References on a separate page (page 12).
3. **Data deposit:** The data have been deposited in the Supporting Information section.
