# Supplementary material for: Curability difference between autochthonous mouse tumors and their transplants in association with immune gene expression
Source: PLoS One. 2026 Apr 24;21(4):e0338289. doi: 10.1371/journal.pone.0338289 (PMC13108875; doi:10.1371/journal.pone.0338289)
Supplement: Attachment — Submitted filename: Response to Reviewers Mar29.docx [file pone.0338289.s007.docx]

**Second Response to Reviewers’ Comment**s (March 29, 2026)

First responses to reviewers comments were described in the Response of Februray 24, 2026.

**Reviewer #1**

Reviewer #1 had no comment.

**Reviewer #2**

Reviewer #2 raised a further question concerning the simultaneous upregulation of Pd1, Pdl1, and Pdl2.

We respond to this question as follows.

“ The co-expression of these genes may appear paradoxical, as they include the inhibitory receptor *Pd1*, its ligands *Pdl1* and *Pdl2*, and T-cell markers *Cd3d* and *Cd8b* associated with anti-tumor immunity. However, the concurrent expression of *Pd1, Pdl1*, and *Pdl2* is not contradictory but rather reflects coordinated activation of immune checkpoint pathways. Such co-expression has been widely reported in cancer and is associated with inflammatory signaling, adaptive immune resistance, and T-cell exhaustion [22, 23]. Notably, co-expression of PD1 and PDL1 has been reported to correlate with favorable postoperative prognosis in human gastric cancer [24]. Furthermore, inflammatory IFNγ feeds back to upregulate expression of immune checkpoint genes [25]. The results of the present study are consistent with these findings. (from page 6, line 168 to page 7, line 177)
